# Tumor blood vessel in 3D reconstruction CT imaging as an risk indicator for growth of pulmonary nodule with ground-glass opacity

**DOI:** 10.1186/s13019-023-02423-x

**Published:** 2023-11-15

**Authors:** Wenfei Xue, Lingxin Kong, Xiaopeng Zhang, Zhifei Xin, Qingtao Zhao, Jie He, Wenbo Wu, Guochen Duan

**Affiliations:** 1 Department of Thoracic Surgery, Hebei Province General Hospital, No. 348, Heping Road West, Xinhua District, Shijiazhuang, 050000 China; 2 Graduate School, Hebei Medical University, Shijiazhuang, 050000 China

**Keywords:** Ground-glass opacity, Persistent malignant pulmonary nodule, Risk factor, GGO growth

## Abstract

**Objective:**

Despite the vital role of blood perfusion in tumor progression, in patients with persistent pulmonary nodule with ground-glass opacity (GGO) is still unclear. This study aims to investigate the relationship between tumor blood vessel and the growth of persistent malignant pulmonary nodules with ground-glass opacity (GGO).

**Methods:**

We collected 116 cases with persistent malignant pulmonary nodules, including 62 patients as stable versus 54 patients in the growth group, from 2017 to 2021. Three statistical methods of logistic regression model, Kaplan–Meier analysis regression analysis were used to explore the potential risk factors for growth of malignant pulmonary nodules with GGO.

**Results:**

Multivariate variables logistic regression analysis and Kaplan–Meier analysis identified that tumor blood vessel diameter (*p* = 0.013) was an significant risk factor in the growth of nodules and Cut-off value of tumor blood vessel diameter was 0.9 mm with its specificity 82.3% and sensitivity 66.7%.While in subgroup analysis, for the GGO CTR < 0.5[C(the maximum diameter of consolidation in tumor)/T(the maximum diameter of the whole tumor including GGO) ratio], tumor blood vessel diameter (*p* = 0.027) was important during the growing processes of nodules.

**Conclusions:**

The tumor blood vessel diameter of GGO lesion was closely associated with the growth of malignant pulmonary nodules. The results of this study would provide evidence for effective follow-up strategies for pulmonary nodule screening.

## Introduction

With the wide utilization of low-dose computed tomography (LDCT), the screening of pulmonary nodules with ground-glass opacity (GGO) is more precise. In a CT window, GGO refers to the increased density inside the lungs, presenting as a foggy or frosty shadow, but not masking the vascular and bronchial texture [1], the lesions with higher density than the lung parenchyma. A series of studies reported that persistent GGO results in the lesions of lung parenchyma and has a high risk of becoming malignant tumors [2, 3]. A long-term pure ground-glass nodule (GGN) is highly associated with early-stage lung adenocarcinoma [4], compared to the solid pulmonary nodules [5, 6].

Based on the LDCT performance, it is better to participate in the CT screening annually for potential patients. Once abnormal symptoms are observed in the scanning windows, such as GGO gray blocks, it is recommended to continue a full-scale examination based on the NCCN or Fleischner Society guidelines in the following several years [7, 8]. Sometimes, the size of malignant GGO does not change for many years [9, 10], whilst some small GGO blocks are continuously growth over time [11]. Therefore, it is important to investigate the risk factors for speeding up GGO growth, which is useful for further effective therapy strategies making.

Early studies reported the clinic-radiological factors for predicting the interval growth of persistent pulmonary nodule through investigating the growth characteristics of these nodules [12–16]. However, there was no one study about the tumor blood vessel of pulmonary nodules up to now. As we know, the tumor progressing rely on the blood vessel invasion [17]. In this study, the pathology confirmed pulmonary nodules with GGO patients were retrospectively collected to investigate the relationship between tumor blood vessel in CT imaging and growth of persistent malignant GGO and to determine the potential influencing factors of GGO for guiding the individual-based precise treatment strategies development.

## Materials and methods

### Patients

In the present study, we collected 116 cased in Hebei General Hospital from January 2015 to May 2021. All these cases are confirmed with pure GGN or part-solid GGN based on the chest thin-section CT with slice thicknesses less than 1.25 mm. Then the cases were divided into two groups, i.e., stable and growing groups by two thoracic surgeons for further study based on the 2011 national lung screening test [18]. In detail, more than 10% increase in nodule diameter was defined as growing GGN, we measured the diameter from coronal, transverse and sagittal view in CT. So the increase of diameter would be selected according to changes of the longest diameter in different views, which were divided into the growing group, and the residual cases belonged to stable group. If for part-solid GGN the diameter was no changed, ≥ 2 mm increase in the solid component should belong to growing GGN. Prior to this study, all 116 patients are periodically examined with the CT in our hospital. During the follow-up period, their GGN shape was tested as well with CT. According to NCCN guidelines, patients with GGN are monitored for a duration of three months before and after this experiment. All the 116 cases did not include the ones only taking chest CT scanning at one time or resection of GGN within three months, nor those any treatment with systemic chemotherapy for concurrent lung cancer. In this study, only the largest GGN blocks/solid component were considered if many GGN were detected in a case CT window. Since the nodule growth interval of some patients was one month, an over of 1 month of the follow-up interval was set for the growing group and the stable group of over 3 months. Patients who could only obtain chest CT data once or resection of GGNs within the initial 3 months were excluded. And patients who received treatment with systemic chemotherapy for concurrent lung cancer also couldn’t be enrolled. For multiple GGN, only the largest GGN was registered. If part-solid GGN and pure GGO exist in one patient, nodules with the largest solid component were selected.

### CT examination

All CT examinations were conducted using dose modulation with the following parameters: 120 kVp, 30–40 or 100–200 mAs, the pitch of 0.75–1.5, and collimation of 0.625–1.25 mm. In order to reveal the morphological features and the relationship between the adjacent bronchi and blood vessels, multiplane reconstruction and 3D volume-rendered images were performed using a picture archiving and communication system (Neusoft Medical Systems, Shenyang, China) and a commercially available 3D reconstruction system (InferRead CT Lung, InferVision, Beijing, China).

**Fig. 1:**
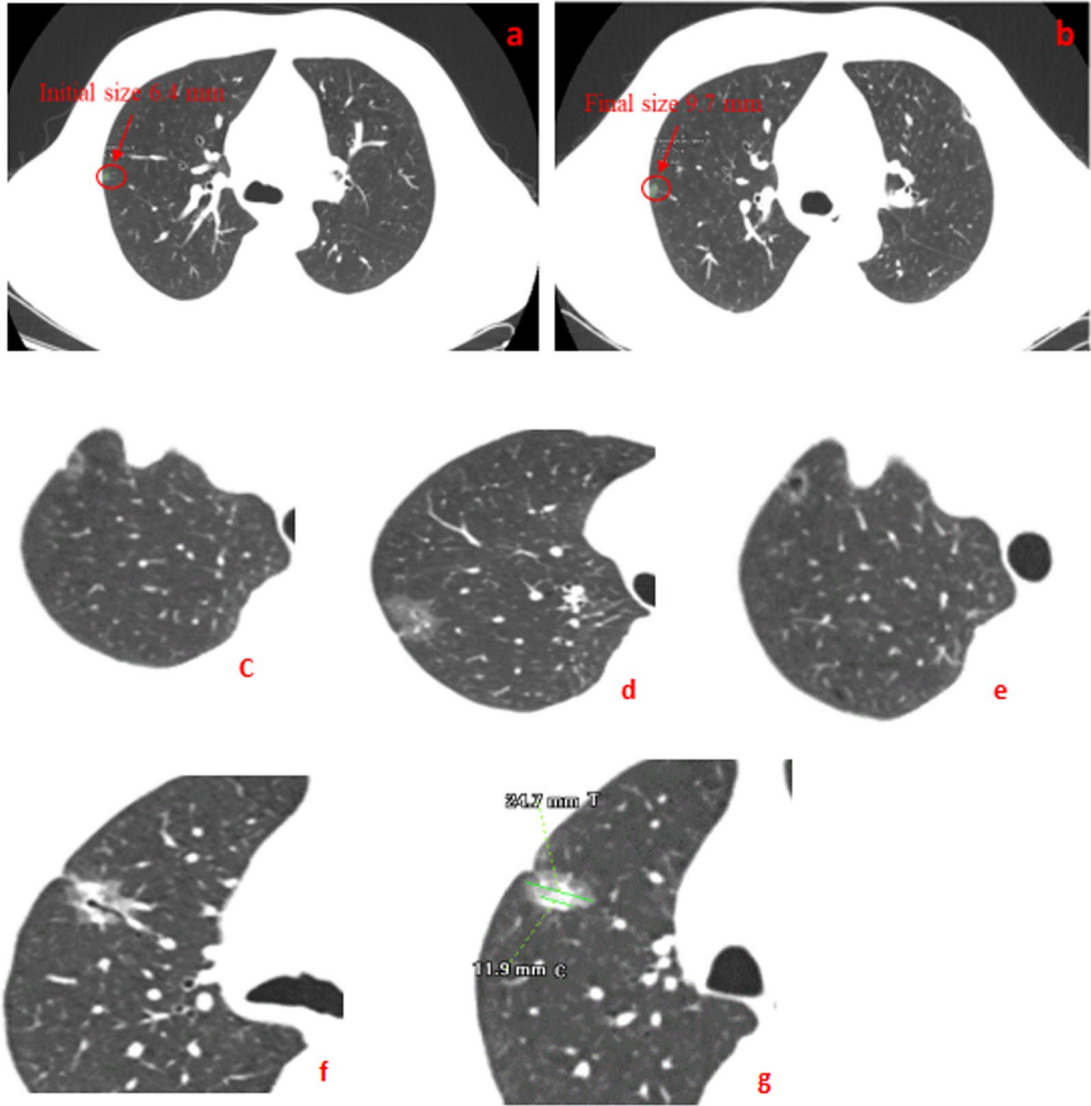
The size change of a GGO from the initial (**a**) to the final period of 3-year (**b**) during this study. (**c**) Pleural indentation sign (**d**) Lobulation sign (**e**) vacuole sign (**f**) A CT imaging included spiculation sign, air bronchus sign and vascular convergence sign, (**g**) The maximum diameter of consolidation in tumor (C) and the greatest diameter of the whole tumor including GGO (T)

**Fig. 2:**
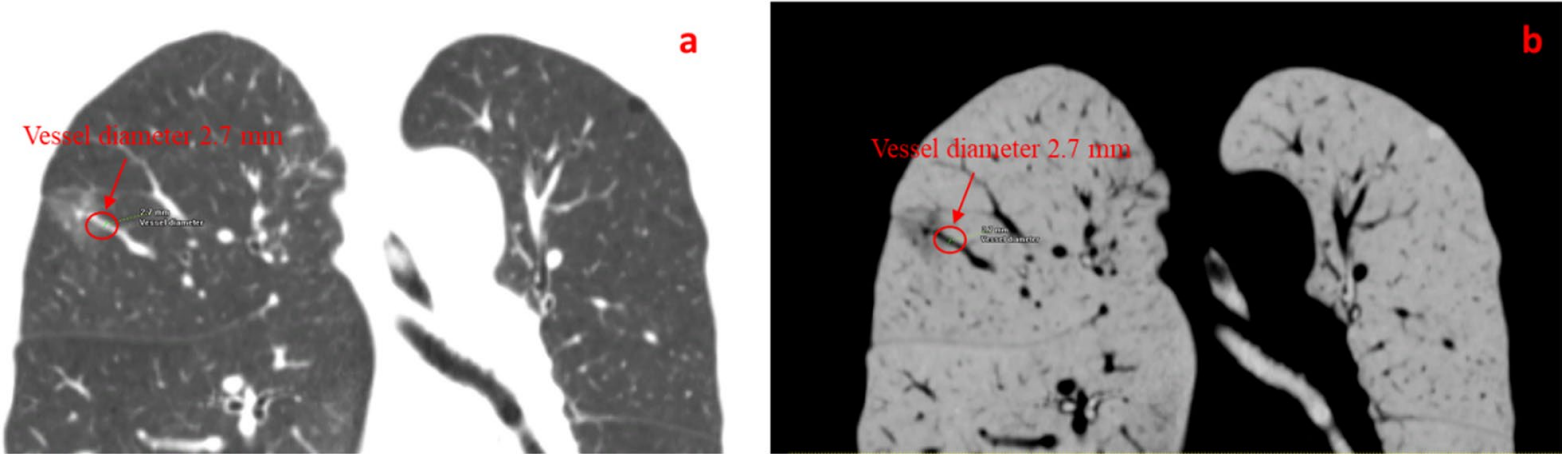
Vessel diameter after entering into the lesion. (**a**) The vessel detected by 3D volume-rendered images and (**b**) the vessel detected with the inverse of lung window

Two board-certified pathologists with 10-year experience of in pathological diagnosis of lung cancer) reviewed the CT images with GGN. They carefully checked the radiology features of both lungs (window width of 1500 HU and window level of 600 HU). The mediastinal windows (width of 500 HU and level of 50 HU) were set as following as in Fig. 1a,b diameter of GGNs (maximal diameter on the axial section) (c) Pleural indentation sign (linear attenuation toward the pleura from GGNs),(d) Lobulation sign, i.e., the appearance of a wavy or scalloped configuration in the lesion’s surface, (e) vacuole sign (single or multiple cystic cavities with a diameter of less than 5 mm in GGNs) (f) Spiculation sign (the presence of strands from the nodule margin into the lung parenchyma, but not reaching the pleural surface); Air bronchus sign (air-filled bronchi in the GGNs); Vascular convergence sign (GGNs with dilated, convergent or tortuous supplying vessels); (g)The maximal diameters of consolidation in tumors (C) and of the whole tumor including GGO (T) in lung window. GGNs were categorized into 2 groups by CTR (C/T ratio) of 0.5; tumor blood vessel diameter (the supplying microvascular with the nodule diameter of larger than 0.5 mm penetrating the nodules. The diameter here was the sum while multiple vessels were observed If there were two vessels, the diameter was the sum of two) as shown in Fig. 2. We measured the value of the vessel and magnified the CT picture by almost 500% which made its precision approach 0.1 mm. The other author rechecked the image interpretation before reaching a consensus. The clinical features of the patients included age, sex, smoking history, family history of tumor, postoperative pathology, degree of infiltration and size of pulmonary nodules. Pathological diagnoses of GGNs after surgically resection were also recorded and classified according to 2015 WHO Classification criteria for lung adenocarcinoma [19]^.^

### Statistical analyses

All statistical analyses were performed using the software SPSS (version 22.0, Inc., Chicago, USA). The differences among the continuous variables of the two groups were compared by using a t-test or Mann–Whitney U test, and the classified data were analyzed by chi-square analysis. The relationship between risk factors and GGN growth was investigated with univariate and multivariate logistic regression analysis and the odds ratio (OR) of 95% confidence interval (95% CI) was calculated. All effects were declared statistically significant at *p* < 0.05. The significant variables in multivariate regression were regarded as potential predictors based on Receiver Operating Characteristic Curve (ROC) analysis. The Youden index was calculated to determine the best cut-off value to maximize sensitivity and specificity with the MedCalc Statistical software (Version 18.2.1). Considering the effects of follow-up time on the accuracy of the results, Kaplan–Meier analyses with the log-rank test were used.

## Results

### Characteristics of study patients

A total of 116 patients with persistent malignant pulmonary nodules (54 in the growing group and 62 in the stable group) were included in this study. The clinical characteristics of the patients in the 2 groups are listed in Table 1. Compared with the stable group, patients in the growing group include a higher fraction of males (53.7% in growing vs 35.5% in stable group, *p* = 0.061) and smokers (37.0% vs 19.4%, *p* = 0.038), and experience of surgical history (42.6% vs 22.6%, *p* = 0.028), and higher patient age (63.4 ± 9.0 in growing group vs 53.8 ± 10.5 in the stable group, *p* < 0.001). The tumor history (*p* = 0.753), tumor initial size (*p* = 0.428), and follow-up duration (*p* = 0.121) were similarly observed in the two groups. After several months of follow-up, all GGNs were confirmed by surgery experts as lung adenocarcinoma. The neoplasm stage (*p* < 0.001) and invasive carcinoma (*p* < 0.001) were significantly different as well between the two groups.

**Table 1:** Patient characteristics and pathological findings in two groups

Characteristics	Growing group (n = 54)	Stable group (n = 62)	*p* values
Age^‡^	63.41 ± 9.03	53.77 ± 10.54	< 0.001
Smoking history			0.038
No	34(63.0%)	50(80.6%)	
Yes	20(37.0%)	12(19.4%)	
Tumor history			0.753
No	48(88.9%)	57(91.9%)	
Yes	6(11.1%)	5(8.1%)	
Gender			0.061
Female	25(46.3%)	40(64.5%)	
Male	29(53.7%)	22(35.5%)	
Invasive			< 0.001
No	27(50.0%)	57(91.9%)	
Yes	27(50.0%)	5(8.1%)	
Surgical history			0.028
No	31(57.4%)	48(77.4%)	
Yes	23(42.6%)	14(22.6%)	
Initial Size(mm)			0.428
< 8	18(33.3%)	23(37.1%)	
8–10	10(18.5%)	17(27.4%)	
10–15	15(27.8%)	15(24.2%)	
> 15	11(20.4%)	7(11.3%)	
Follow up duration (months)			0.121
< 12	14(25.9%)	25(40.3%)	
12–24	12(22.2%)	17(27.4%)	
24–36	14(25.9%)	13(21.0%)	
> 36	14(25.9%)	7(11.3%)	
Stage			< 0.001
0	8(14.8%)	39(62.9%)	
Ia1	17(31.5%)	17(27.4%)	
Ia2	18(33.3%)	5(8.1%)	
Ia3	8(14.8%)	1(1.6%)	
Ib	3(5.6%)	0(0%)	

^‡^Values of the patient age are expressed as the mean ± standard deviation. The data except for the age are the patient populations and the data in the parentheses are the accounting percentage of the total patient population of each group

### Features in CT imaging

In 116 patients, lobulation sign, spiculation sign, pleural indentation, air bronchogram, vessel convergence, vacuole sign were observed in 45 (38.8%), 22 (18.9%), 24 (20.7%), 10 (8.6%), 59 (50.8%), and 41 (35.3%), respectively (Table 2). Compared with the stable group, air bronchogram (16.7% vs 1.6%, *p* = 0.006), vessel convergence sign (70.4% vs 33.9%, *p* < 0.001), vacuole sign (48.1% vs 24.2%, *p* = 0.011), and the median vessel diameter of the GGO nodules (1.25 vs 0, *p* < 0.001); were significant different. However, similar fractions of lobulation sign, spiculation sign, and pleural indentation were observed in the two groups. The diameter of microvascular and the solid component of GGN were significantly different (*p* < 0.001).

**Table 2:** CT characteristics findings in two groups

Characteristics	Growing group (n = 54)	Stable group (n = 62)	*p* values
Vessel diameter (mm)	1.25(0–3.7)	0(0–1.8)	< 0.001
Component			< 0.001
< 0.5	21(38.9%)	55(88.7%)	
> 0.5	33(61.1%)	7(11.3%)	
Lobulation sign			0.059
No	28(51.9%)	43(69.4%)	
Yes	26(48.1%)	19(30.6%)	
Spiculation sign			0.097
No	40(74.1%)	54(87.1%)	
Yes	14(25.9%)	8(12.9%)	
Pleural indentation sign			0.252
No	40(74.1%)	52(83.9%)	
Yes	14(25.9%)	10(16.1%)	
Air bronchus sign			0.006
No	45(83.3%)	61(98.4%)	
Yes	9(16.7%)	1(1.6%)	
Vascular convergence sign			< 0.001
No	16(29.6%)	41(66.1%)	
Yes	38(70.4%)	21(33.9%)	
Vacuole sign			0.011
No	28(51.9%)	47(75.8%)	
Yes	26(48.1%)	15(24.2%)	

The vessel diameters are shown in terms of median and range in the parenthesis

### Risk factors for the growth

Effect of univariate and multivariate variables in patients with growing GGO in (Table 3).A univariable analysis demonstrated that growth of malignant pulmonary nodules was closely associated with patient age (odds ratio [OR], 1.108; 95% confidence interval [CI], 1.057–1.160; *p* < 0.001), gender (OR, 2.109; 95% CI, 1.000–4.448; *p* = 0.050), Smoking history (OR, 2.451; 95% CI, 11.060–5.665; *p* = 0.036), surgical history (OR, 2.544; 95% CI, 1.139–5.680; *p* = 0.023). According to the CT images, the vacuole sign (OR, 2.910; 95% CI, 1.322–6.405; *p* = 0.008), the tumor blood vessel diameter through pulmonary lesions (OR, 4.288; 95% CI, 2.307–7.971; *p* < 0.001), air bronchus sign (OR, 12.200; 95% CI, 1.492–99.785; *p* = 0.020), vascular convergence sign (OR, 4.673; 95% CI, 2.113–10.176; *p* < 0.001), and solid component (OR, 12.347; 95% CI, 4.737–32.185; *p* < 0.001) were also significantly different in the patients of two groups, indicating these are the risk factors. In a multivariable analysis, the patient age (OR, 1.065; 95% CI, 1.004–1.131; *p* = 0.037), the solid component of GGN (OR, 11.38; 95% CI, 3.004–43.107; *p* < 0.001), the vacuole sign (OR, 6.542; 95% CI, 1.943–22.025; *p* = 0.002), and the tumor blood vessel diameter through pulmonary nodules (OR, 2.933; 95% CI, 1.260–6.825; *p* = 0.013) were closely related to the growth of persistent malignant pulmonary nodules.

**Table 3:** Effects of patient and CT imaging characteristics on the GGN growth based on univariate and multivariate analysis

Variables	Univariate			Multivariate		
	OR	95% CI	*p* values	OR	95% CI	*p* values
Age	1.108	1.057–1.160	< 0.001	1.065	1.004–1.131	0.037
Gender	2.109	1.000–4.448	0.05			
Initial Size	1.067	0.982–1.159	0.127			
Smoking history	2.451	1.060–5.665	0.036			
Surgical history	2.544	1.139–5.680	0.023			
Tumor history	1.425	0.409–4.961	0.578			
Lobulation sign	2.102	0.984–4.490	0.055			
Spiculation sign	2.362	0.905–6.171	0.079			
Pleural indentation sign	1.82	0.732–4.522	0.197			
Air bronchus sign	12.2	1.492–99.785	0.02			
Vascular convergence sign	4.673	2.113–10.176	< 0.001			
vacuole sign	2.91	1.322–6.405	0.008	6.542	1.943–22.025	0.002
Component	12.347	4.737–32.185	< 0.001	11.38	3.004–43.107	< 0.001
Vessel diameter	4.288	2.307–7.971	< 0.001	2.933	1.260–6.825	0.013

*CI* confidence interval; *GGN* ground-glass nodule; *OR* odds ratio;

### Analysis in terms of time to growth

**Fig. 3:**
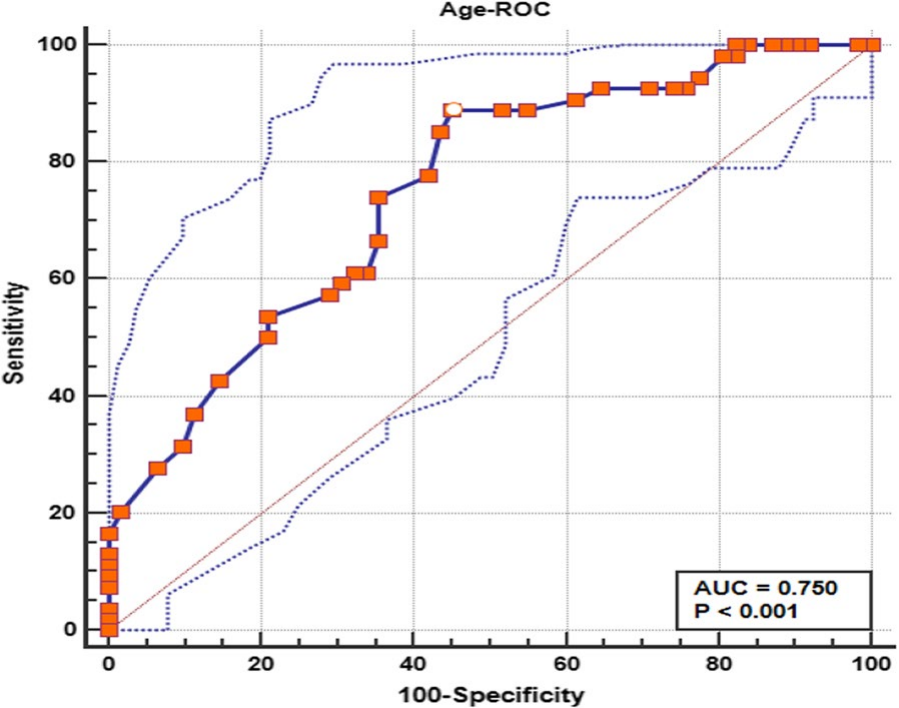
The ROC analysis of patient age showing that the cut-off value was 54.0 with sensitivity of 0.889 and 1-specificity of 0.548

**Fig. 4:**
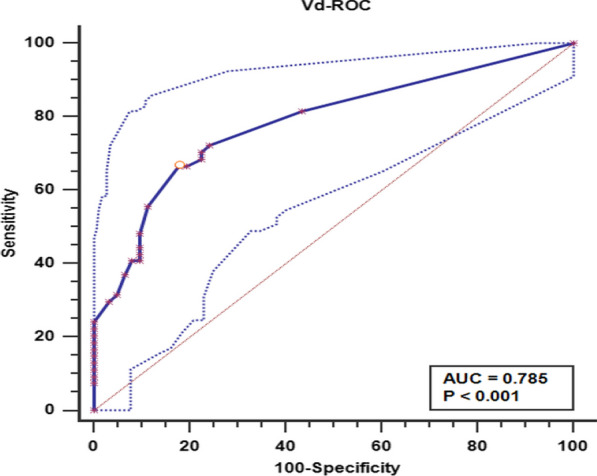
The ROC analysis of patient age showing that the vessel diameter cut-off point was 0.9 mm

**Fig. 5:**
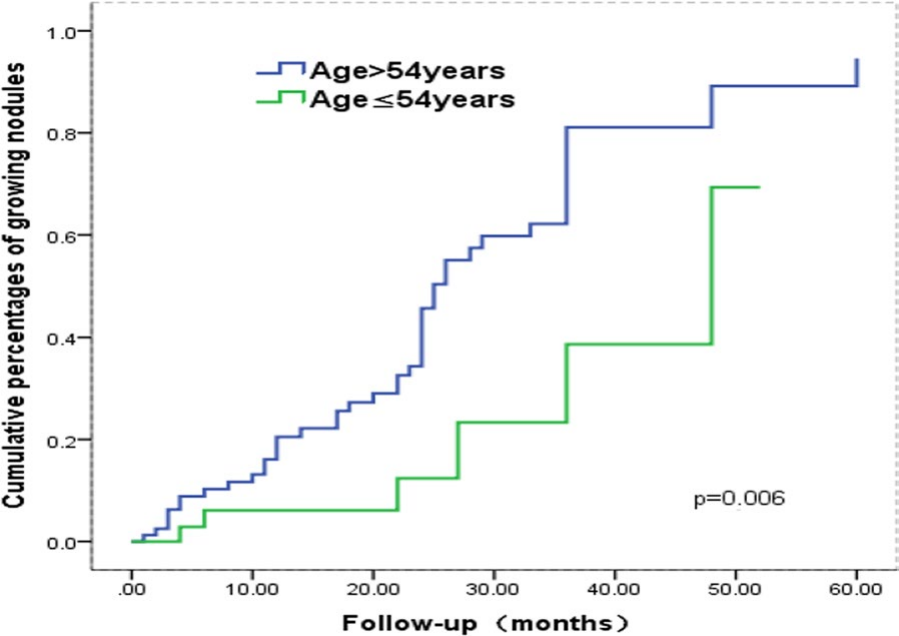
The variation in the cumulative percentages of growing nodules of two patient age groups with the increasing follow-up time

**Fig. 6:**
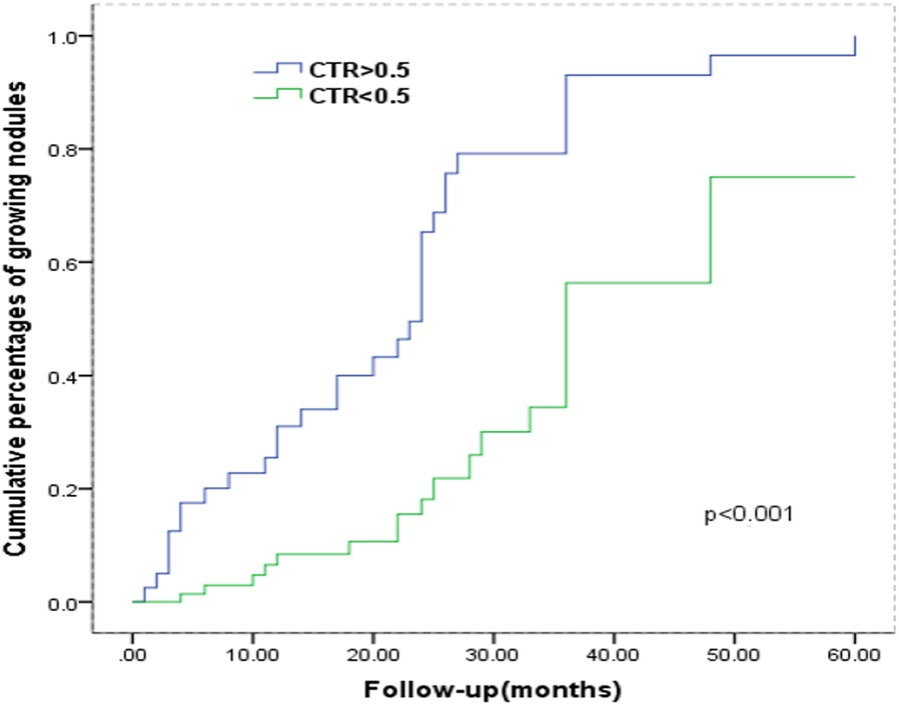
Kaplan–Meier plot for time to nodule growth according to CTR (the maximum diameter of consolidation in tumor (C) and the maximum diameter of the whole tumor including GGO (T). The CTR means C/T ratio. Part-solid GGNs (CTR > 0.5) show significantly higher cumulative percentages of growth than GGNs with CTR < 0.5

**Fig. 7:**
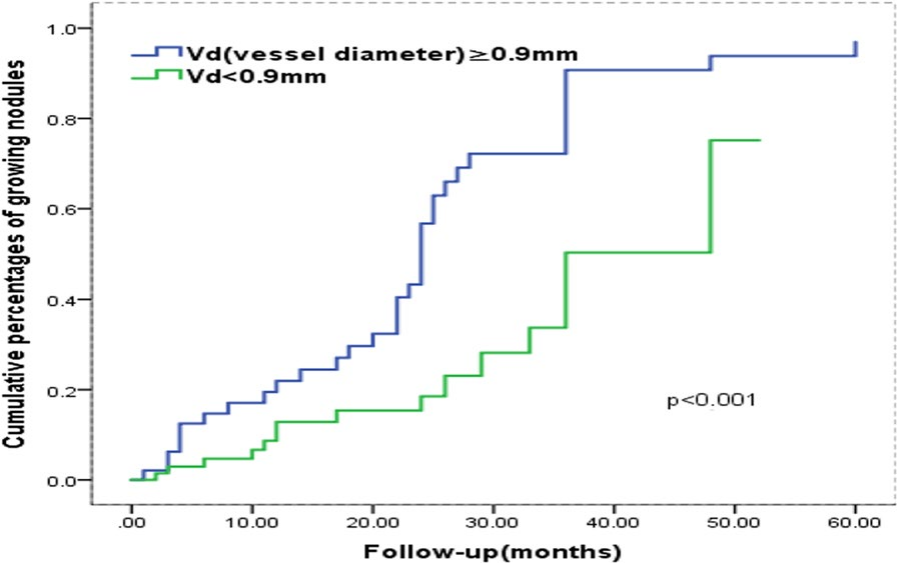
Kaplan–Meier plot for time to nodule growth according to the vessel diameter entering the GGN. those with Vd ≥ 0.9 mm growth much faster than those with Vd < 0.9 mm

A ROC analysis was carried out to determine the dividing point of the patients’ age and the size of pulmonary nodules vessel (Table 4). A positive correlation was observed between the patient age and vessel diameter of both groups. Specifically, patient age (AUC 0.750, *p* < 0.001) and vessel diameter (AUC 0.785, *p* < 0.001) were significantly effective. Results showed that the age of 54-year old is crucial for the nodule growth The cut-off value between Growing group and Stable group patients of patient age score was found to be equal to 54.0 with a sensitivity of 0.889 and specificity of 0.548 (Fig. 3) and the vessel diameter cut-off point of 0.90 with sensitivity of 0.667 and specificity of 0.823 (Fig. 4). According to Kaplan–Meier analysis, patient age (*p* < 0.001; Fig. 5), solid component of GGN (*p* < 0.001; Fig. 6) and the diameter of vessel penetration in GGN (*p* < 0.001; Fig. 7) were significantly different as well between two groups.

**Table 4:** Identification of the characteristics of growing and stable pulmonary nodules based on the ROC index

	AUC	Sensitivity	Specificity	*p* values	95% CI	Cut-off value	Yd
Age	0.75	0.889	0.548	< 0.0001	0.661–0.826	54	0.437
Vd	0.785	0.667	0.823	< 0.0001	0.699–0.856	0.9	0.489

*AUC* area under curve; *CI* confidence interval; *Vd* vessel diameter; *Vs* vacuole sign. *Yd* Youden index

### Subgroup logistic analysis for GGO( CTR < 0.5).

Because the solid component was the main risk factor for growth, the analysis of the subgroup was necessary for the GGO with a solid component ratio < 0.5 (Table 5). A univariable analysis demonstrated that growth of GGO was closely associated with patient age (odds ratio [OR], 1.082; 95% confidence interval [CI], 1.020–1.148; *p* = 0.009), For the CT images, the vacuole sign (OR, 4.333; 95% CI, 1.498–12.532; *p* = 0.007), the main tumor blood vessel diameter through GGO lesions (OR, 3.570; 95% CI, 1.571–8.108; *p* = 0.002). Multivariable analysis showed the vacuole sign (OR, 4.272; 95% CI, 1.266–14.420; *p* = 0.019), and the vessel diameter through pulmonary nodules (OR, 2.561; 95% CI, 1.116–5.879; *p* = 0.027) were the risk factors for the growth.

**Table 5:** Subgroup analysis of effects of patient and CT imaging characteristics on the GGN(CTR < 0.5) growth

Variables	Univariate			Multivariate		
	OR	95% CI	*p* values	OR	95% CI	*p* values
Age	1.082	1.020–1.148	0.009	1.055	0.987–1.128	0.114
Gender	1.235	0.429–3.558	0.695			
Initial size	0.967	0.835–1.119	0.967			
Smoking history	1.120	0.340–3.684	0.852			
Tumor history	0.638	0.067–6.057	0.695			
Lobulation sign	1.129	0.415–3.581	0.719			
Spiculation sign	1.361	0.308–6.024	0.685			
Pleural indentation sign	1.143	0.266–4.906	0.857			
Air bronchus sign	2.700	0.161–45.247	0.490			
Vascular convergence sign	2.032	0.726–5.691	0.177			
Vacuole sign	4.333	1.498–12.532	0.007	4.272	1.266–14.420	0.019
Vessel diameter	3.570	1.571–8.108	0.002	2.561	1.116–5.879	0.027

*CI* confidence interval; *GGN* ground-glass nodule; *OR* odds ratio

## Discussion

In this study, multivariate analysis results showed that the solid component of nodules was an independent predictor of GGO growth. However, some studies [20–22], pointed out that there was no relationship between the solid component and the growth of pulmonary nodules, but the nodule size was an independent factor influencing the growth of pulmonary nodules. In fact, the expansion of solid components increases the invasiveness of the lesions [23, 24]. We investigated the pathology of invasive adenocarcinoma in two groups and there was a significant difference between the growing and stable groups in terms of tumor stages (*p* < 0.001). The reason for this discrepancy was mainly due to the unknown of the GGNs’ pathology during the follow-up duration.

The most difficult process of this study was the sample collection, i.e., the patients with GGN. This is mainly because of the uncertainty of the pulmonary nodule growth and aggravation. So far, there have not been any guidelines depicting the risk factors of GGN growth [25, 26]. It is necessary to identify the influencing factors of nodule growth in clinical practices.

The results of multivariate analysis in the present study showed that patient age, vacuole sign, the solid component of GGN and vascular diameter significantly affected the nodule growth. In addition, Kaplan–Meier analysis with the log-rank test showed similar results. Cho et al. [24] analyzed 218 patients with ground glass nodules of the lung and found that older patient (≥ 65 years old) was an independent factor associated with subsequent GGN growth. However, a threshold of 54-year old was observed in this study. This different result is because only the patients with malignant nodules were included in this study. Previous reports showed that lesion diameter is a predictor of GGO growth [9, 10], while there was no difference in it between the two groups in this study because of the different sizes of GGN in these studies.

A series of studies reported that the appearance of pleural indentation, lobulation and spiculation signs is a signal of a higher degree of malignancy [27, 28]. But few studies reported the relationship between CT imaging features and the growing GGNs. In this study, compared with the stable group, there was a statistical difference in terms of air bronchogram (*p* = 0.006), vessel convergence sign (*p* < 0.001), vacuole sign (*p* = 0.011), and the median vessel diameter of the GGO nodules (*p* < 0.001) in the growing group. Similarly, the vacuole signs and the vessel diameters were significantly different between the two groups based on the multivariate analysis. The pathological basis of the vacuole sign consists of two parts [29]: (1) the normal or emphysematous lung tissue appearance in the nodule can speed up the alveolar emphysema expansion due to the contraction of the scar tissue in the nodule, eventually resulting in low-density vesicles; (2) the necrotic lung tissue, i.e., a small amount of excretion or necrotic tissue dehydration, can shrink the volume of vacuoles. The air bronchus sign was observed while accompanied by the pulmonary artery entering the tumors. Previous studies reported that vacuole signs and air bronchogram signs are not only malignant evidence of GGN but also signs of infiltration degree of lung adenocarcinoma [30–33]. Therefore, the appearance of the vacuole signs is a direct indicator of tumor growth and continuously monitoring benefits the timely and effective intervention.

The vascular convergence sign of tumors is crucial to determining the nature of a pulmonary nodule [34]. Tumor biology research showed that continuous angiogenesis and vascular remodelling are indicators of tumor development in the early stage [35]. The detection of abnormal vessels is of great value as well to estimate the malignant potential of GGN [34, 36–38]. To date, there are few studies to report the importance of using vascular convergence signs and vascular diameter to predict GGN growth. In this study, the results of both multivariate and Kaplan–Meier analysis showed that vascular diameter was a useful independent predictor of GGN growth and the larger the vascular diameter showed the higher the probability of nodular growth, with the sensitivity of 0.667 and the specificity of 0.823.

There are still some limitations in this study. For example, the sample size is small and the measurement of blood vessel sizes is prone to man-made errors, especially for the tiny ones. Because highly selective study population of the malignant pulmonary nodules with GGO,the selective bias existed in this study. Moreover, in the current study, the effects of chest CT scanning on the evaluation results of GGN are indefinite. Therefore, further studies are needed to be conducted and more reliable and quantitative methods are needed to be developed to effectively extract the features from the CT images to improve the prediction accuracy of the occurrence and growth of GGN.

In general, the identification of GGN growth is a complicated task. However, the pulmonary nodules growth with GGO can be predicted by considering the clinical responses and morphological features in CT images, such as parents’ age and the diameter of tumor blood vessels. The obtained results are significant for the further clinical decision making on the management of pulmonary nodules induced by GGN.

## Data Availability

Not applicable.
